# Long-term Changes in Extreme Air Pollution Meteorology and the Implications for Air Quality

**DOI:** 10.1038/srep23792

**Published:** 2016-03-31

**Authors:** Pei Hou, Shiliang Wu

**Affiliations:** 1 Atmospheric Sciences Program, Michigan Technological University, Houghton, MI, 49931, USA; 2 Dept. of Geological and Mining Engineering and Sciences, Michigan Technological University, Houghton, MI, 49931, USA; 3 Dept. of Civil and Environmental Engineering, Michigan Technological University, Houghton, MI, 49931, USA

## Abstract

Extreme air pollution meteorological events, such as heat waves, temperature inversions and atmospheric stagnation episodes, can significantly affect air quality. Based on observational data, we have analyzed the long-term evolution of extreme air pollution meteorology on the global scale and their potential impacts on air quality, especially the high pollution episodes. We have identified significant increasing trends for the occurrences of extreme air pollution meteorological events in the past six decades, especially over the continental regions. Statistical analysis combining air quality data and meteorological data further indicates strong sensitivities of air quality (including both average air pollutant concentrations and high pollution episodes) to extreme meteorological events. For example, we find that in the United States the probability of severe ozone pollution when there are heat waves could be up to seven times of the average probability during summertime, while temperature inversions in wintertime could enhance the probability of severe particulate matter pollution by more than a factor of two. We have also identified significant seasonal and spatial variations in the sensitivity of air quality to extreme air pollution meteorology.

Besides affecting the mean values of various meteorological variables, a critical implication of climate change is to alter the frequency and intensity of a suite of extreme meteorological events^1^,^2^,^3^,^4^,^5^,^6^,^7^. Some of these extreme events such as heat waves, temperature inversions and atmospheric stagnation episodes have important implications for atmospheric chemistry and air quality^8^,^9^,^10^,^11^,^12^,^13^. There have been many studies on the potential impacts of climate change on air quality^14^,^15^,^16^,^17^,^18^,^19^,^20^,^21^,^22^,^23^,^24^,^25^,^26^,^27^,^28^,^29^,^30^, but most of those analyses have generally focused on the impacts associated with the changes in the average meteorological conditions (such as temperature, humidity, precipitation, etc.). The long-term evolution of extreme air pollution meteorology on the global scale and the potential impacts on air quality have not been investigated.

We first examine the evolution of extreme air pollution meteorology in the past six decades. We follow the World Meteorological Organization method^31^ on the definition of heat waves with some modification - A heat wave is defined when the daily maximum temperature at a given location exceeds the “climatological” daily maximum temperature (averaged over the reference period of 1961–1990) by at least 5 K for more than two consecutive days. Fig. 1a shows the average annual occurrences of heat waves in the first 30-year (1951–1980) period as well as the percentage changes when compared with the more recent 30-year (1981–2010) period. Significant increases in heat waves in the more recent decades are observed over most continental regions, especially the high latitude regions. For most regions, the trends in the frequency of heat waves are similar to those identified in the literature^31^. It is noticeable that the frequency of heat waves have decreased over some areas in the United States in the past decades. The annual average frequency of heat waves for the global non-polar continental regions is found to increase by 25.8 ± 3.3% (Table 1). The largest increases (around 40%) are found during Northern Hemisphere spring (March-May) and summer (June-August) seasons.

For temperature inversions, we examine the atmospheric temperature profile below 800 hPa which is most relevant to air quality. A temperature inversion event is defined when the temperature at a higher level is at least 0.1 K higher than the temperature below. On a global scale, a general increase in the occurrences of temperature inversions is found, except over the high latitudes (Fig. 1b). A warmer climate is expected to increase the evapotranspiration, releasing more latent heat in the upper troposphere which could reduce the temperature lapse rate in the troposphere, especially over the tropics and mid-latitude regions. As a consequence, the atmospheric stability is generally expected to increase with climate change leading to more temperature inversions. On the other hand, the decreases in temperature inversions over polar regions reflect the strong surface warming there in the past decades, partly driven by the positive feedback associated with snow/ice albedo^32^. For non-polar continental regions in the Northern Hemisphere, the trends in temperature inversion events show clear seasonal variations: the strongest increases are observed in summer (by 17.4 ± 7.0%) while little changes are found in winter.

The definition of atmospheric stagnation used in this study follows the National Climatic Data Center (NCDC) methodology^33^ with a relative threshold to focus on the local changes: A stagnation episode is defined when the 10 m wind speed, 500 hPa wind speed, and precipitation at a given location are all less than their climatological values for the reference period (1961–1990) by at least 20%. Figure 1c shows that the occurrences of atmospheric stagnation episodes have increased over most continental areas. Our results are consistent with Wang *et al*.^34^ who studied the changes in atmospheric stagnation episodes over the U.S. region during the past decades. For non-polar continental regions, the annual average atmospheric stagnation events have increased by 4.5 ± 0.8%. This increase is partly due to the weakening of surface winds driven by climate change^35^. In addition, the more intense but less frequent precipitation in a warmer climate could also contribute to the increased frequency of atmospheric stagnation events^36^.

To examine the impacts on air quality from each specific extreme meteorological event (heat waves, temperature inversions or atmospheric stagnation episodes), we analyze air quality data from the U.S. EPA AQS database for 2001–2010 together with the meteorology data for the same period. The air quality data are processed into the same spatial resolution as the meteorology data (2.5° × 2.5°) by averaging the available data from all the sites within the same grid cell. Daily average concentrations for PM_2.5_ and afternoon (1–4 pm local time) average concentrations for ozone (derived from hourly ozone data) are used in the analysis. We first compare the average air quality on “event days” with those on “non-event days”. The statistical significance of the differences between these two groups are evaluated with t-tests with a 95% confidence interval. Figure 2a shows the percentage change of seasonal average afternoon ozone concentrations on days with heat waves compared to those on days without heat waves for each season. The highest sensitivity of surface ozone to heat waves is found during summer and fall. The low sensitivity in winter and spring reflects the weaker photochemical ozone production in those seasons^37^,^38^. From Fig. 2a we can also see large spatial variations in the sensitivity of ozone to heat waves: The strongest sensitivities are found in the eastern United States and the west coast, where the mixing ratios of afternoon ozone are enhanced by more than 40% on days with heat waves, reflecting the strong emissions of ozone precursors^39^ and hence high ozone production there. As discussed above, the frequency of heat waves have decreased in the past decades over some areas in the United States (Fig. 1a), which could have cancelled out some of the increases in high ozone pollution risk induced by other factors over those areas in the past decades.

We find that heat waves have much stronger impacts on air quality than single “hot” days with the same temperature. Figure 3 shows the response of summer ozone concentrations to temperature, one group for all days in the season, another only for days with heat waves. We can see that with the same temperature, ozone concentrations on days with heat waves are significantly higher than those non-consecutive “hot” days, especially over the 293–313 K temperature range. Generally, the ozone concentrations on days with heat waves are more than 4.5 ppb higher than those projected by the average ozone-temperature correlation. This reflects the build-up effects from the extended period of high temperature during heat wave events. On the other hand, the “heat wave effects” appear weaker when the temperature is above 313 K (Fig. 3). In comparison, Steiner *et al*.^13^, based on observational data from California, reported that the daily maximum ozone is most sensitive to temperature in the range of 295–312 K but the ozone formation is suppressed when the temperature is above 312 K.

The impacts of temperature inversions on seasonal average concentrations of PM_2.5_ are shown in Fig. 2b. The strongest impacts from temperature inversions are observed in winter time with daily average PM_2.5_ concentrations enhanced by 40% or more over large areas in the United States. The impacts are much weaker in summer and fall, mainly limited to the northeast and northwest states. In contrast, significant impacts on PM_2.5_ concentrations associated with atmospheric stagnation episodes are found for all seasons throughout the United States (Fig. 2c), with the largest increases in PM_2.5_ concentrations exceeding 40% over large areas.

We further examine the impacts of extreme air pollution meteorology on the cumulative probability distributions of ozone and PM_2.5_ concentrations (Fig. 4). For each season, the cumulative probability distributions of ozone mixing ratios for days with heat waves were compared with those without heat waves (Fig. 4a). We can see that extreme air pollution meteorology usually has the greatest impacts on the high end of the distributions, which represents the high pollution episodes. For example, during summer time, the 95^th^ percentile ozone is increased by about 25% while the 50^th^ percentile ozone is only increased by about 19% due to heat waves. Similar feature is found for the impacts on PM_2.5_ from temperature inversions and atmospheric stagnation episodes. In winter time, the 95^th^ percentile PM_2.5_ concentration is increased by 65% while the 50^th^ percentile PM_2.5_ concentration only increases by 28% in response to temperature inversions (Fig. 4b). Similarly, atmospheric stagnation episodes are found to have little effects on the low end of PM_2.5_ distributions (which represent the clean conditions) but significant impacts on the high pollution episodes for each season (Fig. 4c).

For a specific air pollutant (i.e. ozone or PM_2.5_), we define the high pollution days as the top 10% most polluted days for each season and examine their sensitivities to various extreme air pollution meteorological events. To better quantify the impacts from extreme events on high pollution episodes and their relative importance, we define an impact factor as the enhancement in the probability of high pollution episodes due to extreme meteorological events (see Methods section for details). The impact factors for high ozone pollution days in summer associated with the three types of extreme events on state level are shown in Fig. 5a,c and similarly the impact factors for different regions in the United States are shown in Fig. 5b,d.

We find that the heat wave is the most important meteorological event in leading to high ozone pollution days in summer for most areas in the United States (Fig. 5a,b). The impact factors for ozone pollution associated with heat waves are particularly high in the eastern United States (such as Louisiana, Alabama and Georgia), with values up to 6, which indicates the probability of severe ozone pollution would be enhanced by a factor of 7 when there are heat waves over those areas. The large spatial variations in the impact factors reflect the regional variations in anthropogenic and natural emissions of air pollutants and their precursors, climate, orography and geography (such as whether downwind or upwind of major air pollutant source regions). The highest impact factors for temperature inversions are found over the eastern United States and the Northwest region, while the highest impact factors for atmospheric stagnation episodes are found over the Midwest.

Figure 5c,d show the impact factors for PM_2.5_ in winter associated with the three types of extreme events. The highest impact factors (up to 1.6) are found for temperature inversions over the western regions. The impact factors for atmospheric stagnation episodes are generally higher in the eastern United States, and consistently positive (indicating positive correlation between stagnation episodes and high PM_2.5_ pollution episodes) throughout the United States. In contrast, some negative impact factors are found for heat waves. One likely reason is the decrease of ammonium nitrate (a major component of PM_2.5_ in winter time) at higher temperatures. In addition, during warmer days in winter, there would be less residential biomass burning, which is a major source for aerosols in the Western United States^40^. This could also contribute to the negative correlation between heat waves and PM_2.5_ in winter.

For the locations with extreme meteorological events identified, we find that on average there are about one third of the times (32% as shown in Table S1) with more than one extreme events occurring simultaneously. To account for the interactions between different types of extreme meteorological events and their synthetic effects on air quality, we also calculate the impact factors for high pollution days associated with multiple events occurring simultaneously. The impact factors for U.S. high ozone and PM_2.5_ days in different seasons are summarized in Table 2. With the increase in the number of simultaneously occurring extreme events (from 0–3), the probability of high pollution episodes almost always increases (with the notable exception of the winter season). The highest impact factor (3.3) is found for summer ozone associated with the combination of three extreme events. This implies that, on average over the whole United States, the probability of high ozone pollution would be enhanced by more than a factor of 4 compared to the seasonal average when the three extreme events occur at the same time in summer.

## Methods

We first examine the evolution of extreme air pollution meteorology in the past six decades based on the National Centers for Environmental Prediction (NCEP) reanalysis dataset^41^. The dataset covers the 1951–2010 period with a horizontal resolution of 2.5° latitude by 2.5° longitude and a temporal resolution of 6 hours (http://www.esrl.noaa.gov/psd/). To identify the long-term changes in extreme air pollution meteorology (heat waves, temperature inversions and atmospheric stagnation episodes), we compare the climatological data for extreme events for two 30-yr periods: 1951–1980 vs. 1981–2010. We also conduct further analyses to examine the sensitivity of our results to the metrics for definition/identifying air pollution meteorological events and datasets used (see more details in Supplementary Information). To quantify the impacts of extreme air pollution meteorology on air quality, we analyze air quality data (focusing on ozone and PM_2.5_) from the U.S. Environmental Protection Agency (EPA) AQS (http://www.epa.gov/airdata/) database for 2001–2010 together with the meteorology data for the same period. The air quality data are processed into the same spatial resolution as the meteorology data (2.5° × 2.5°) by averaging the available data from all the sites within the same grid cell. Daily average concentrations for PM_2.5_ and afternoon (1–4 pm local time) concentrations for ozone (derived from hourly ozone data) are used in the analysis.

For each grid cell, we classify the air quality data into various groups based on the meteorological conditions at the same time (e.g. heat wave group vs. no heat wave group). If any group contains less than 3 valid air quality data, we exclude that cell from the corresponding analysis.

To compare the relative importance of various extreme air pollution meteorological events in leading to high pollution episodes for various regions, we carry out further analysis focusing on the high pollution days, which are defined as the top 10% most polluted days for that season during the 2001–2010 period at that location. For days with a specific meteorological event (heat waves, temperature inversions, or stagnation episodes) occurring, we calculate the probability of those days falling in the top 10% high pollution days (i.e. having the top 10% highest concentrations for a given pollutant – ozone or PM_2.5_ in this case). This probability (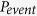) is then compared with the average probability (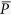) for all days during the same season (whether or not it has any extreme meteorological event) falling into the top 10% high pollution days (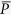 should be equal to 10% following the definition). We also define an impact factor (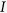) for a specific meteorological event as


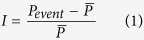


where


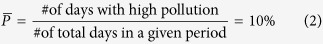



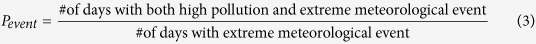


We use the impact factor to quantify the impacts of extreme air pollution meteorology on high pollution episodes. It clearly shows the changes in the probability of severe air pollution associated with certain extreme air pollution meteorological events.

We note that the U.S. anthropogenic emissions declined during the 2001–2010 period which has important implications for air quality. But we do not expect these emission changes to have any significant impacts on the derived sensitivities of air quality to extreme air pollution meteorology since a) our derived air quality sensitivities to extreme meteorology are expressed as relative (percentage) changes; and b) the 10-yr period is a relatively short time window in the context of global climate change therefore we expect the climate-induced changes in extreme air pollution meteorology should be small during this period. We have performed additional analyses to confirm that our derived sensitivities are not affected by emission changes (see Supplementary Information for more details).

## Additional Information

**How to cite this article**: Hou, P. and Wu, S. Long-term Changes in Extreme Air Pollution Meteorology and the Implications for Air Quality. *Sci. Rep*. **6**, 23792; doi: 10.1038/srep23792 (2016).

## Supplementary Material

Supplementary Information

## Figures and Tables

**Figure 1:**
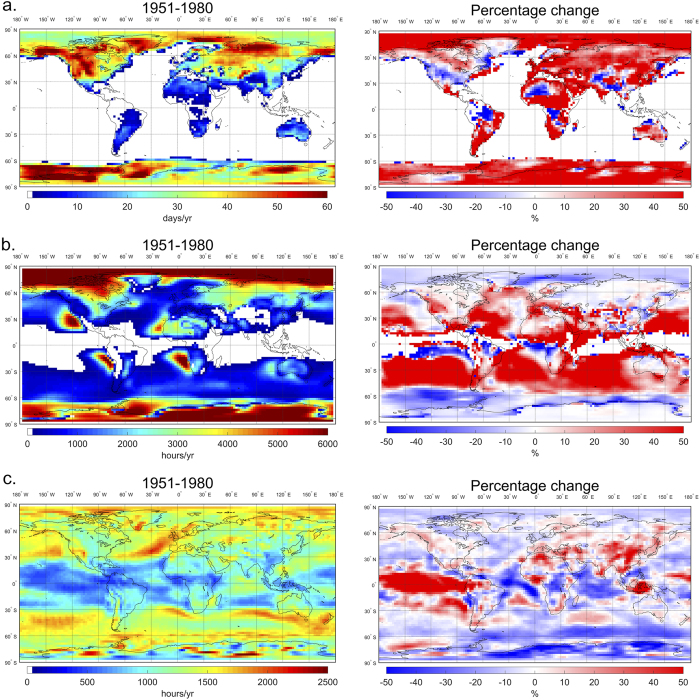
Changes in the frequency of extreme air pollution meteorological events in the past six decades (based on the NCEP reanalysis data): (a) heat waves (days/yr); (b) temperature inversions (hrs/yr); (c) atmospheric stagnation episodes (hrs/yr). Left: 1951–1980 average; right: percentage change (%) between 1951–1980 and 1981–2010. (Map is generated with coarse coastline built in MATLAB R2014b [URL: http://www.mathworks.com/products/matlab/]).

**Figure 2:**
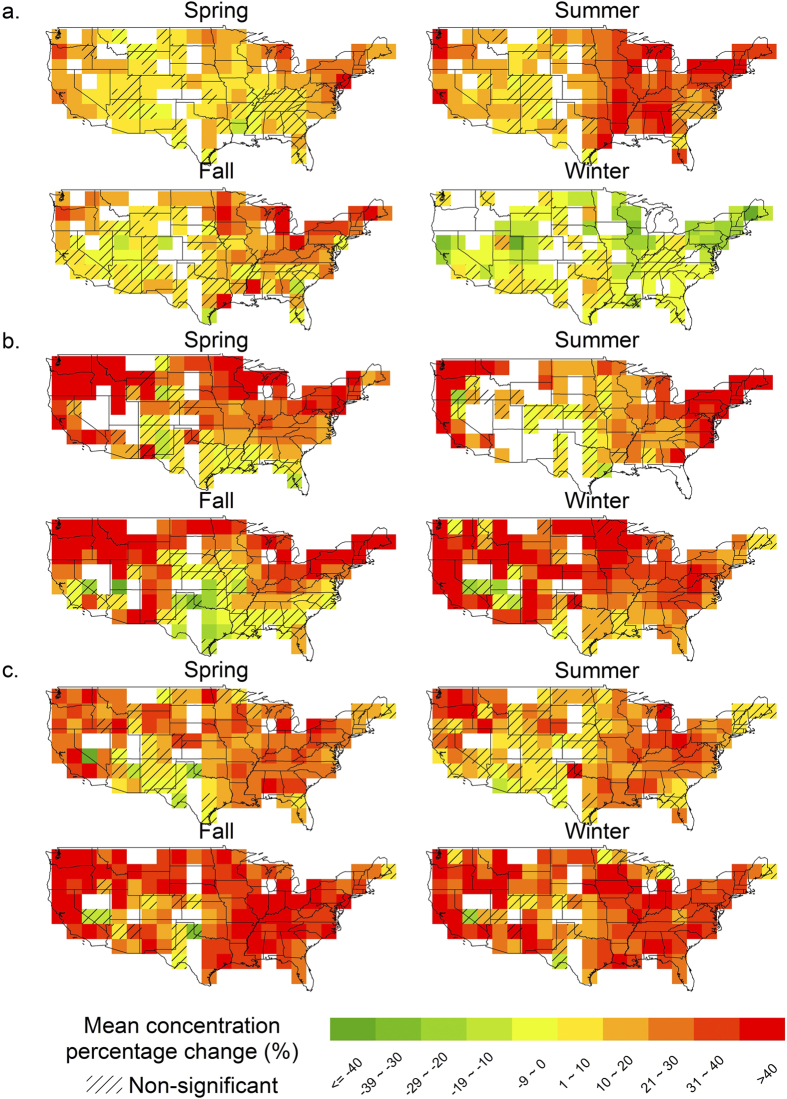
Enhancements in the seasonal average air pollutant concentrations by extreme meteorological events. Shown as the percentage change (%) of mean concentrations (for either ozone or PM_2.5_) on days with a specific meteorological event (event groups) compared to those on days without that event occurrence (no-event groups): (**a**) ozone vs. heat waves; (**b**) PM_2.5_ vs. temperature inversions; (**c**) PM_2.5_ vs. atmospheric stagnation episodes. Shadowed regions indicate that the differences between the two groups are statistically non-significant at the 95% confidence interval. Blank regions indicate those with less than 3 data points for either group. (Map is generated with ArcGIS 10.2.2 [URL: http://www.esri.com/software/arcgis/arcgis-for-desktop]).

**Figure 3:**
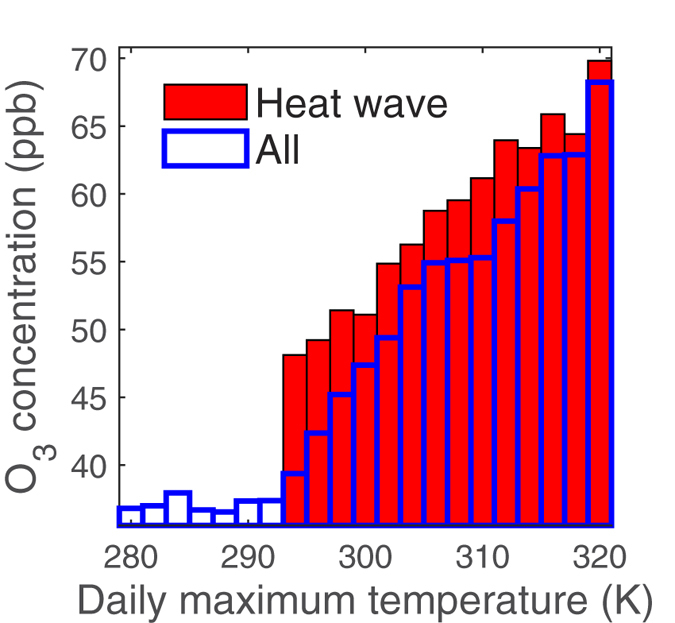
Summer ozone concentrations as a function of daily maximum temperature based on 2001–2010 data in the United States. Blue curve shows the average ozone concentrations for all the days with temperature falling in specific temperature bins while the red curve only covers days with heat waves.

**Figure 4:**
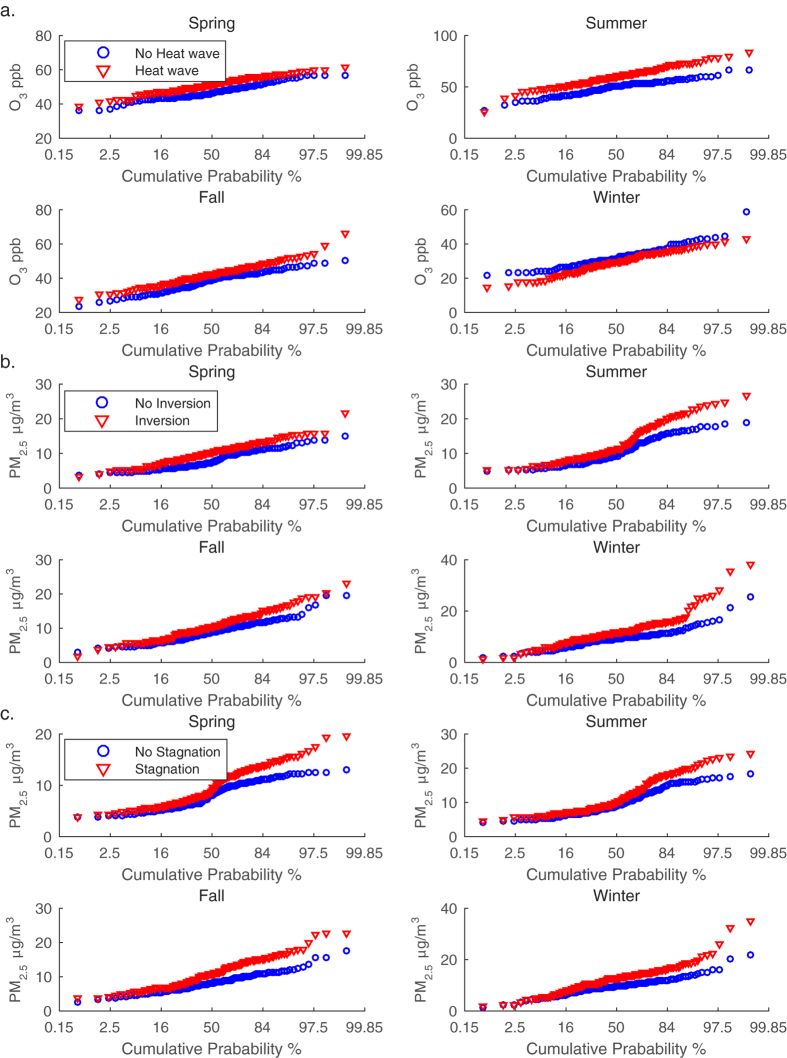
Cumulative probability plots for concentrations of air pollutants. Red triangle: event group; blue circle: no-event group. (**a**) ozone mean concentrations of heat wave group and no heat wave group; (**b**) PM_2.5_ mean concentrations of temperature inversion group and no temperature inversion group; (**c**) PM_2.5_ mean concentrations of atmospheric stagnation group and no atmospheric stagnation group.

**Figure 5:**
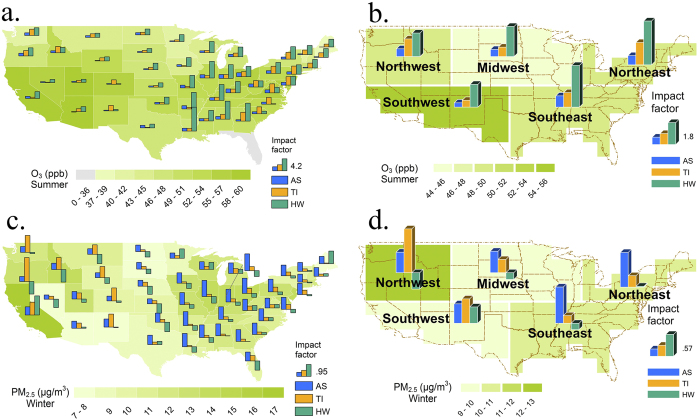
Enhancements in the probability of high pollution episodes by extreme air pollution meteorological events for different states and regions in the United States. Shown as the impact factor for (**a**) summer ozone by state; (**b**) summer ozone by region; (**c**) winter PM_2.5_ by state; and (**d**) winter PM_2.5_ by region associated with various meteorological events (heat waves, temperature inversions and atmospheric stagnation episodes; indicated by the green, orange and blue bars respectively). The impact factor is defined as the enhancement in the probability of high pollution episodes due to extreme meteorological events. Background color indicates the mean concentration for that pollutant. Bar plots for the 4 smallest states (includes District of Columbia, Rhode Island, Delaware and Connecticut) are omitted to increase accessibility. (Map is generated with ArcGIS 10.2.2 [URL: http://www.esri.com/software/arcgis/arcgis-for-desktop]).

**Table 1:** The percentage change ± s.e.m. (%) in the average frequencies of extreme events (HW: heat waves; TI: temperature inversions; AS: atmospheric stagnation episodes) for the global non-polar continental regions between the two 30-yr periods: 1981–2010 vs. 1951–1980.

Event	Season	Global	Northern Hemisphere
HW	Annual	25.8 ± 3.3	24.5 ± 3.1
	March-May	45.4 ± 4.3	44.9 ± 4.1
	June-August	40.3 ± 5.0	40.9 ± 5.3
	September-November	9.9 ± 3.6	6.5 ± 3.7*
	December-January	17.9 ± 3.5	16.2 ± 3.3
TI	Annual	6.2 ± 3.2	6.7 ± 3.4*
	March-May	9.1 ± 3.9	9.0 ± 4.3
	June-August	10.3 ± 5.3*	17.4 ± 7.0
	September-November	8.8 ± 3.6	10.6 ± 3.9
	December-January	1.8 ± 3.4*	1.6 ± 3.0*
AS	Annual	4.5 ± 0.8	6.8 ± 0.9
	March-May	7.2 ± 0.9	9.8 ± 1.1
	June-August	6.7 ± 1.1	11.8 ± 1.3
	September-November	3.6 ± 0.9	5.5 ± 1.0
	December-January	0.5 ± 0.9*	1.0 ± 1.1*

‘*’ indicates statistically non-significant results at the 95% confidence interval.

**Table 2:** The impact factor for high pollution days (ozone and PM_2.5_) over the United States associated with various extreme meteorological events (None: no event; HW: only heat waves; TI: only temperature inversions; AS: only atmospheric stagnation episodes; All: three kinds of events happened at the same time).

Species	Season	None	HW	TI	AS	HW&TI	HW&AS	TI&AS	All
O_3_	Spring	−0.5	0.1	0.0	0.0	1.2	1.0	1.1	3.0
	Summer	−0.5	1.2	0.4	0.2	3.0	2.1	1.3	3.3
	Fall	−0.5	−0.1	−0.2	0.4	0.8	0.8	0.6	2.1
	Winter	0.1	−0.4	−0.1	0.2	−0.1	0.1	0.1	0.0
PM_2.5_	Spring	−0.4	0.0	0.1	0.2	0.6	0.7	0.8	1.9
	Summer	−0.3	0.8	0.2	0.2	1.9	1.2	0.7	2.5
	Fall	−0.4	0.2	−0.3	0.4	1.0	1.0	0.5	2.0
	Winter	−0.5	−0.7	−0.1	0.2	−0.2	0.3	1.2	0.8

High pollution days are defined as the top 10% most polluted days for each season during 2001–2010. The impact factor is defined as the enhancement in the probability of high pollution episodes due to extreme meteorological events.
