# Supplementary material for: Long-term Changes in Extreme Air Pollution Meteorology and the Implications for Air Quality
Source: Sci Rep. 2016 Mar 31;6:23792. doi: 10.1038/srep23792 (PMC4815017; doi:10.1038/srep23792)
Supplement: Supplementary Information [file srep23792-s1.doc]

# Supplementary Information

**Title:**

**Long-term Changes in Extreme Air Pollution Meteorology and the Implications for Air Quality**

**Authors and affiliations:**

Pei Hou^a,b^, and Shiliang Wu^*a,b,c^

^a^Atmospheric Sciences Program, Michigan Technological University, Houghton, MI, 49931, USA.

^b^Dept. of Geological and Mining Engineering and Sciences, Michigan Technological University, Houghton, MI, 49931, USA.

^c^Dept. of Civil and Environmental Engineering, Michigan Technological University, Houghton, MI, 49931, USA.

^*^ To whom correspondence should be addressed: [slwu@mtu.edu](mailto:slwu@mtu.edu)

We have applied various metrics for identifying each air pollution meteorological event and do not find significant impacts on our results. For stagnation episodes, we use different definitions, including a) If the 10 m wind speed, 500 hPa wind speed, and precipitation at a given location are all less than their climatological values for the reference period (1961-1990) by at least 20%. (the one currently being used in the manuscript); b) If the 10 m wind speed, 500 hPa wind speed, and precipitation at a given location all fall in the lowest 20% when compared to the distributions of these variables for the reference period (1961-1990). They lead to similar results (on both the spatial variations and temporal trends in stagnation events), so we finally settle on the one currently used in the MS which appears most straightforward for the readers to understand. Similarly, we have tried various definitions for heat waves such as exceeding the climatological value by certain degrees or by certain percentage, and there were little difference in the identified trends.

We have also compared the NCEP reanalysis data with the MERRA data [1] for cross referencing. Due to limit of computing resources, we have only been able to process 10 years of MERRA data (1981-1985 and 2006-2010). We compare results from these two datasets based on 5-year averages: 2006-2010 vs. 1981-1985. Fig. S1 shows the side-by-side comparison of the spatial distributions of extreme air pollution meteorological events identified based on these two datasets. The frequency of extreme air pollution meteorological events over different continental regions are summarized in Table S2. In most cases, analyses based on these two databases show the same direction in long-term trends. However, significant differences between these two databases are identified for some regions (e.g., heat waves over South America; temperature inversions over the Southern Oceans). This reflects the uncertainties associated with these databases that we are not able to quantify in this study. On the other hand, it is reassuring to see that these two datasets are generally consistent in identifying the trends of extreme air pollution meteorology over continental regions where we have concerns of air pollution (30°N-60°N).

We also carry out additional analysis by using the MERRA data instead of the NCEP data to examine the impacts on air quality from extreme air pollution meteorology. As we can see from Fig. S2 and Fig. S3, there two datasets shows essentially the same sensitivity of air quality (for both ozone and PM) to extreme meteorological events for 2006-2010.

There have been significant changes in the anthropogenic emissions of ozone and PM_2.5_ precursors during the 2001-2010 period (detailed information available from the U.S. EPA - <https://www.epa.gov/air-emissions-inventories/air-pollutant-emissions-trends-data>). These changes could affect the derived sensitivity of air quality to extreme meteorological events if there has been any significant trends in air pollution meteorology during the same period and these trends correlate with the emission change. However, the 10-year period is a relatively short time frame in the context of global climate change so we expect the climate-induced changes in extreme air pollution meteorology are small during this period. Therefore we do not expect the emission changes to have any significant impacts on the derived sensitivities when the sensitivities are expressed as the relative (percentage) changes. Nevertheless, we have carried out two additional tests to further confirm that the derived sensitivities are not affected by emission changes.

In the first test, we separate the 10-yr data into 2 groups of 5-yr data (2001-2005 and 2006-2010 respectively). We found the sensitivities of air quality to extreme meteorological events derived based on these two groups are very close and they do not show any significant differences. For example, in summer, the enhancement in the mean concentration of ozone due to heat waves is around 20% for both groups (19.84% for the 2001-2005 group and 20.36% for the 2006-2010 group).

For the second test, we processed the air quality data into “detrended” data to eliminate the effects from emission changes. We apply a least square linear regression to the original air quality data

$y_{i}^{'}=\alpha+\beta t_{i}$ (S1)

where $\alpha$ and $\beta$ are coefficients of the linear regression and $y_{i}^{'}$ is the “trend data” for air quality at time $t_{i}$. The detrended air quality $d_{i}$ is calculated as

$d_{i}=y_{i}-y_{i}^{'}+\bar{y}$ (S2)

where $y_{i}$ is the original air quality data at time $t_{i}$ and $\bar{y}$ is the average of original air quality data. When we compare the enhancements in air pollution by extreme events derived based on the original air quality data to those derived based on the detrened data, we find they are essentially the same. For example, for the enhancement in summer ozone due to heat waves, we find that the results only differ by 0.51% and for summer PM_2.5_ they only differ by 0.05%. Therefore we believe these two tests further confirm that the emission changes during this period do not have any significant impacts on the derived sensitivities of air quality to extreme air pollution meteorology.

**Reference:**

[1]. Rienecker, M. M. *et al*. MERRA: NASA's Modern-Era Retrospective Analysis for Research and Applications. *J. Climate*. **24**, 3624-3648(2011).

|  | 1981-1985 averages | | Percentage changes between the two periods (2006-2010 vs. 1981-1985) | |
| --- | --- | --- | --- | --- |
|  | NCEP | MERRA | NCEP | MERRA |
| HW | 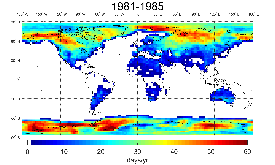 | 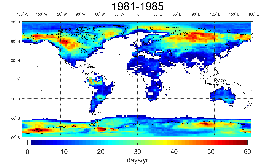 | 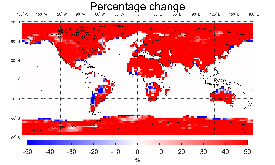 | 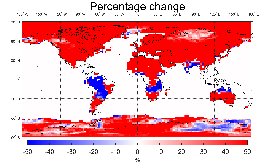 |
| AS | 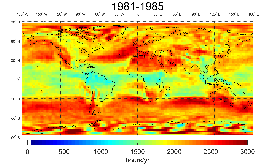 | 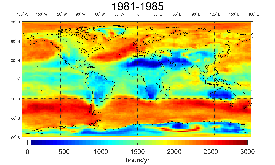 | 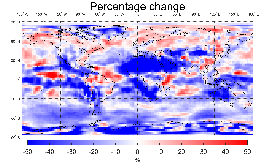 | 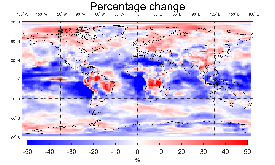 |
| TI | 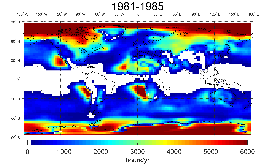 | 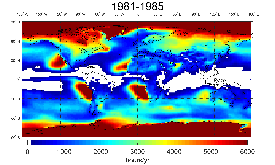 | 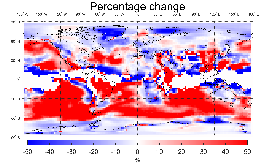 | 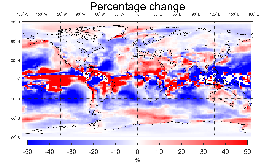 |

**Fig. S1.** Long-term trends (2006-2010 vs. 1981-1985) in extreme air pollution meteorology based on the NCEP reanalysis compared with the MERRA data (HW: heat waves; TI: temperature inversions; AS: atmospheric stagnation episodes). (Map is generated with coarse coastline built in MATLAB R2014b [URL: http://www.mathworks.com/products/matlab/]).

| NCEP | MERRA |
| --- | --- |
| 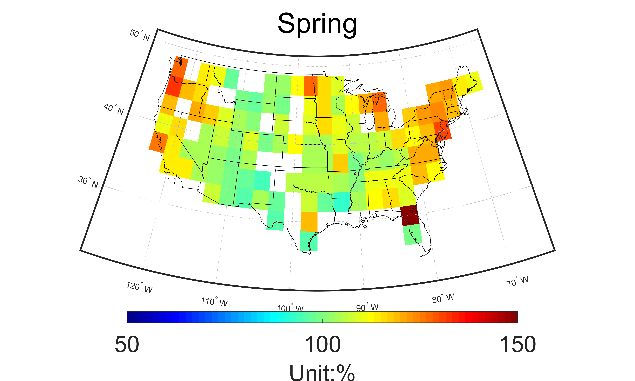  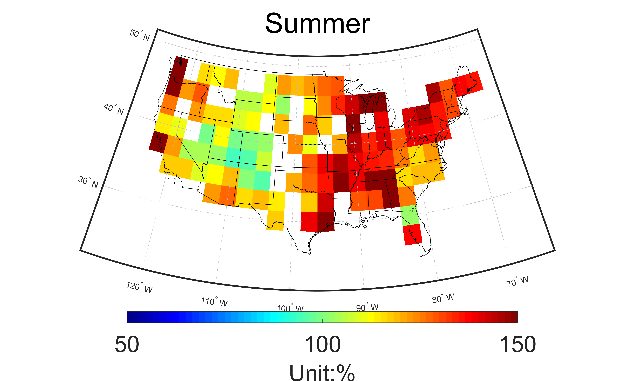  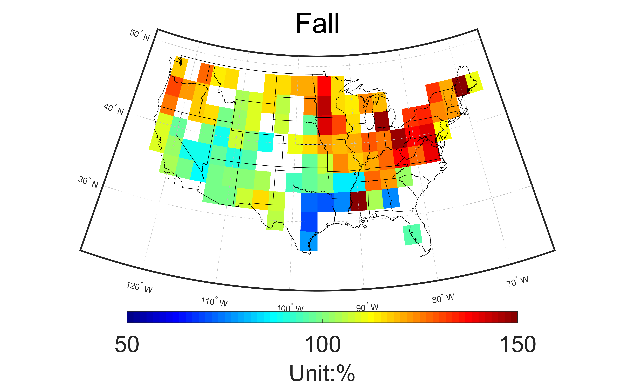  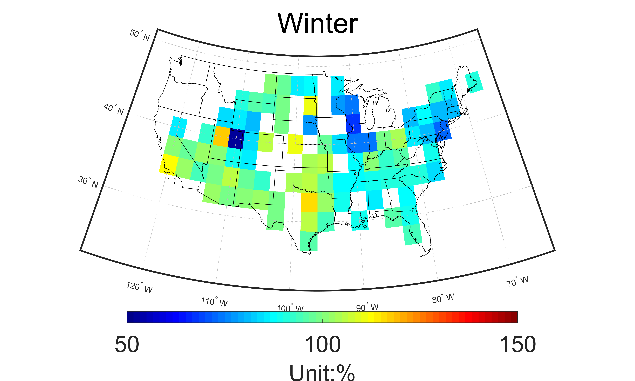 | 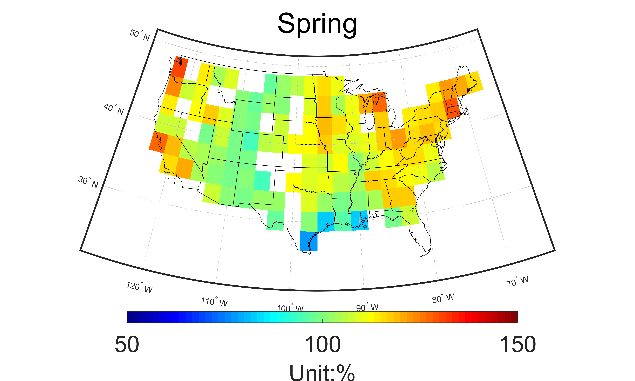  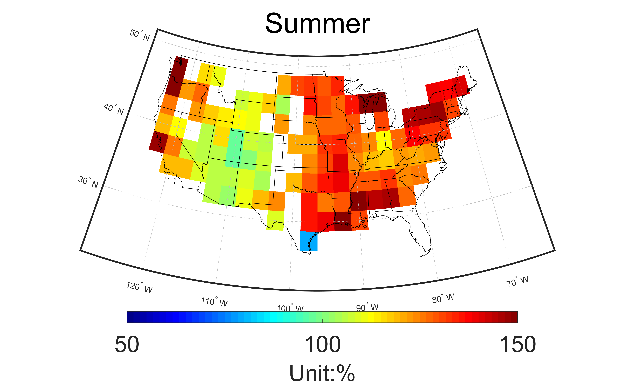  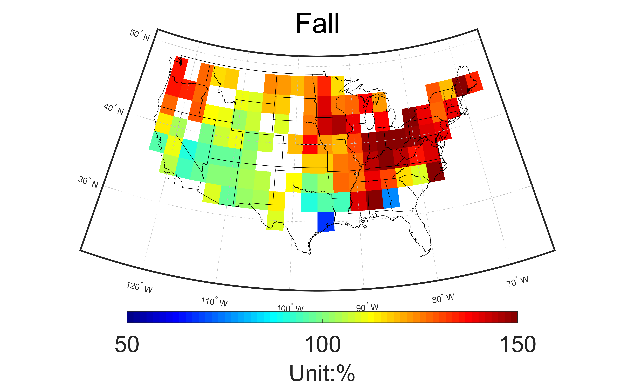  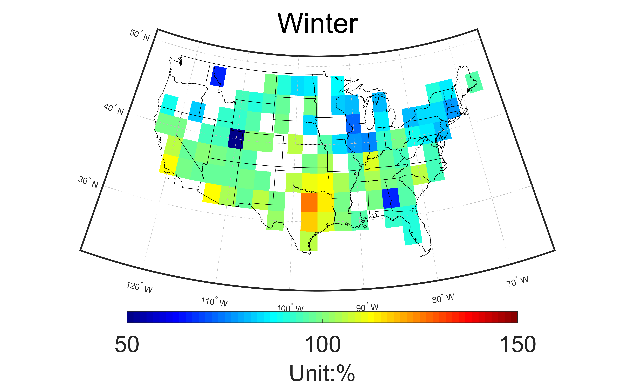 |

**Fig. S2.** Impacts of heat waves on ozone in the United States (shown as the ratio of ozone concentrations on days with heat waves and those without heat waves) based on NCEP vs MERRA data (2006-2010). (Map is generated with U.S. state polygon boundaries built in MATLAB R2014b [URL: http://www.mathworks.com/products/matlab/]).

| NCEP | MERRA |
| --- | --- |
| 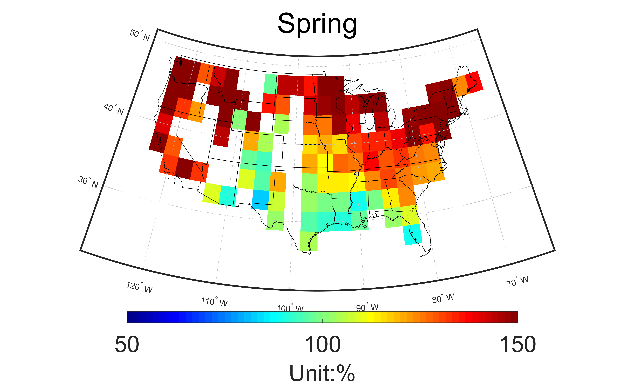  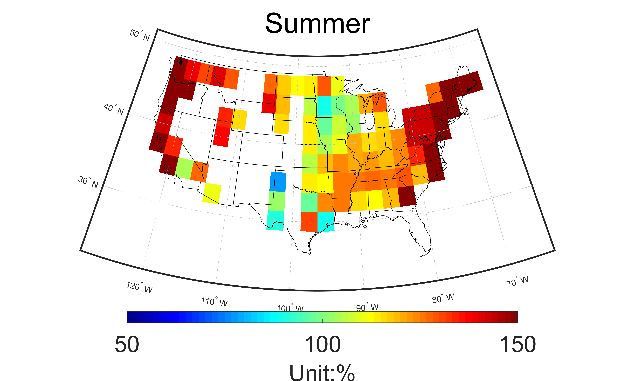  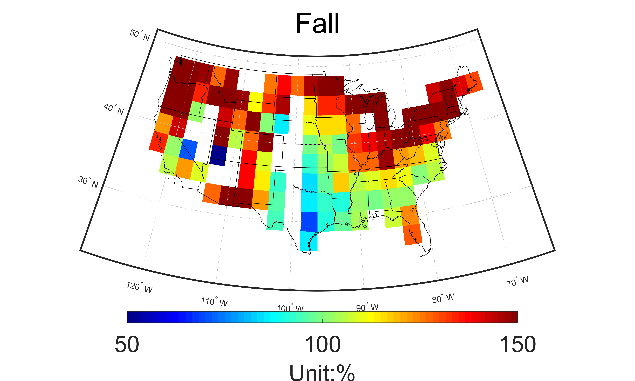  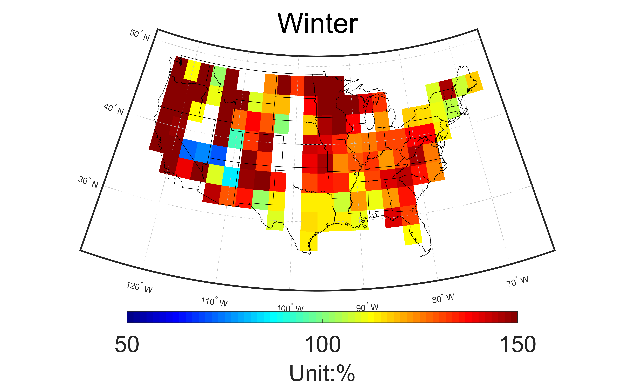 | 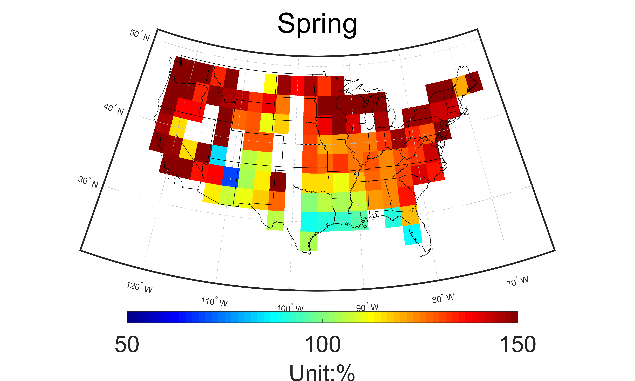  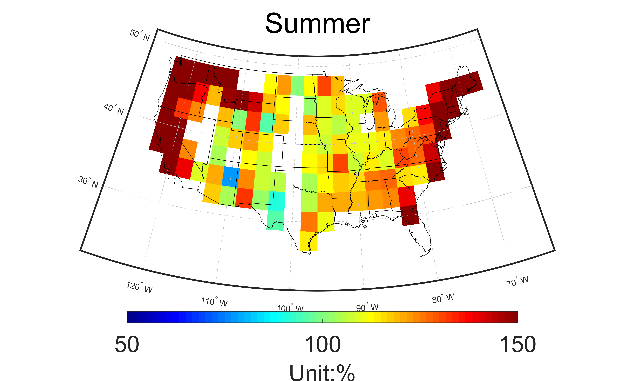  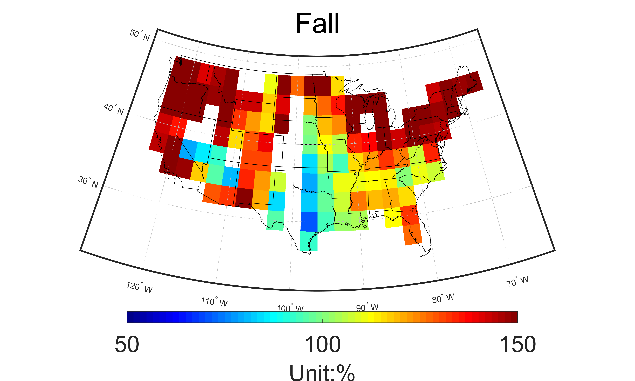  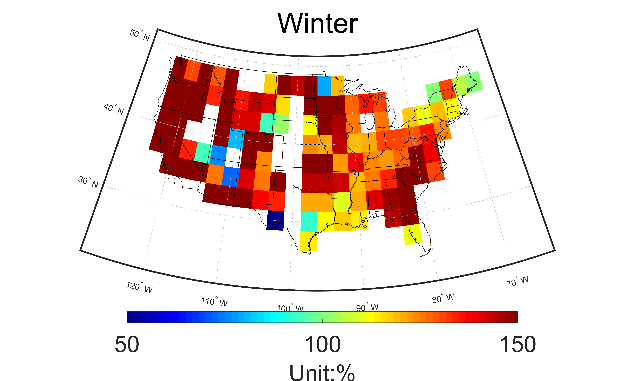 |

**Fig. S3.** Impacts of temperature inversions on PM_2.5_ in the United States (shown as the ratio of PM_2.5_ concentrations on days with temperature inversions and those without temperature inversions) based on NCEP vs MERRA data (2006-2010). (Map is generated with U.S. state polygon boundaries built in MATLAB R2014b [URL: http://www.mathworks.com/products/matlab/]).

**Table S1**. Number of extreme air pollution meteorological events identified over the U.S. region for the 2001-2010 period (HW: heat waves; TI: temperature inversions; AS: atmospheric stagnation episodes).

|  | None | Single event | | | Multiple events | | | |
| --- | --- | --- | --- | --- | --- | --- | --- | --- |
|  |  | Only HW | Only TI | Only AS | HW&TI | HW&AS | TI&AS | All |
| number | 38349 | 3048 | 14274 | 13753 | 1902 | 2688 | 8151 | 1794 |
| total | 38349 | 31075 | | | 14535 | | | |

**Table S2**. The percentage change ± s.e.m. (%) in the annual average frequencies of extreme events (HW: heat waves; TI: temperature inversions; AS: atmospheric stagnation episodes) for the long-term trends (2006-2010 vs. 1981-1985) in different continental regions based on the NCEP reanalysis data compared with the MERRA data (*indicates statistically non-significant results at the 95% confidence interval).

| Regions | Data | HW | TI | AS |
| --- | --- | --- | --- | --- |
| 90°N-60°N | NCEP | 95.9 ± 3.9 | -6.7 ± 2.4 | 0.9 ± 1.7* |
|  | MERRA | 105.9 ± 8.4 | -1.8 ± 2.5* | 6.2 ± 2.8 |
| 60°N-30°N | NCEP | 195.5 ± 6.3 | 0.6 ± 3.1* | -10.2 ± 0.9 |
|  | MERRA | 201.9 ± 13.8 | -1.3 ± 2.1* | -1 ± 1.1* |
| 30°N-0° | NCEP | 321 ± 25.7 | -0.9 ± 6.9* | -36.4 ± 1.2 |
|  | MERRA | 425.3 ± 68.4 | 2.5 ± 6.6* | -16.1 ± 1.3 |
| 0°-30°S | NCEP | 158.2 ± 21 | -0.9 ± 8.1* | -24 ± 1.1 |
|  | MERRA | 107.1 ± 29.2 | -9.5 ± 6.3* | -20.1 ± 1.0 |
| 30°S-60°S | NCEP | 84.8 ± 18.7 | 3.1 ± 11.2* | -25.8 ± 2.3 |
|  | MERRA | 98.9 ± 34.1 | -7.9 ± 1.9 | -5.1 ± 0.8 |
| 60°S-90°S | NCEP | 107.2 ± 6.6 | -5.3 ± 3.0* | -32.7 ± 2.1 |
|  | MERRA | 29.5 ± 4.8 | -0.3 ± 2.5* | -4.7 ± 3.5* |
